# The positioning of women nurses as ‘risky’ in United Kingdom suicide prevention policy documents: a critical policy analysis

**DOI:** 10.3389/fsoc.2026.1730850

**Published:** 2026-02-23

**Authors:** Hilary Causer, Anna Conolly, Barbara Howard-Hunt, Chinenye Anetekhai, Carrie-Ann Black, Elaine Scott, Ruth Riley

**Affiliations:** 1 School of Health Sciences, University of Surrey, Guildford, Surrey, United Kingdom; 2 School of Nursing and Midwifery, Birmingham City University, City South Campus, Birmingham, United Kingdom; 3 South London and Maudsley NHS Foundation Trust, Maudsley Hospital, London, United Kingdom; 4 Nottingham Healthcare NHS Foundation Trust, Nottingham, United Kingdom

**Keywords:** discrimination, feminist methods, nurse suicide, policy, policy analysis, suicide, women nurses

## Abstract

**Introduction:**

Women nurses in the global north more likely to die by suicide than women in other occupations. Current suicide research is largely quantitative and individualises and pathologises nurses. Suicide prevention policy echoes this approach, focusing on individualised risk factors, thereby missing the opportunity to explore contextual, systemic and workplace factors that may contribute to suicide in women nurses. This critical policy analysis explores how distress, suicidality and suicide prevention in women nurses is positioned and constructed in policy and with what political, social and personal consequences.

**Methods:**

A critical intersectional feminist design was adopted to interrogate the data and draw out issues pertinent to women nurses. This work was co-produced with women nurses. Bacchi’s ‘What’s the problem represented to be?’ method of critical policy analysis to inform the data extraction and analysis. We employed a feminist perspective and adapted Lazar’s five principles of feminist discourse praxis. Documents were sourced from governmental and organisational websites and via search engines and were screened against our inclusion criteria. Data was extracted to inform an overview of included documents and for the critical analysis.

**Results:**

Nine documents met our inclusion criteria. We found some stark silences in the included documents regarding suicide in women nurses, and in the health services. Suicide is positioned as a problem of risky people, and as a workforce, rather than a workplace issue. Three narratives were developed to convey the core findings of the analysis: Invisible nurses and silenced suicide; People as risky; Responsibilising the workforce. Four themes sit within ‘Responsibilising the workforce’: Nurses as risky; Knowledge and means; Workforce problems; Workforce solutions.

**Conclusion:**

Current policy documents engage a language of risk which pathologises and responsibilises individuals and minoritised groups as causing high rates of suicide within communities and health workplaces. The impact of socio-economic, political and systemic contexts is overlooked as shaping the lives of suicidal people.

## 1 Introduction

Globally, more than 720,000 people die by suicide every year (WHO, 2025). In the United Kingdom (UK) there was an average of 6,417 deaths by suicide per year between 2012 and 2022 (NCISH, 2025), with men being three times more likely to die by suicide than women. Research approaches in suicidology have focused largely on individual risk factors (White, 2017), taking a psychocentric view of suicidality in terms of theory, policy and practice (Marsh, 2020), whereby human problems are seen as pathological and individual (Rimke, 2016). Such a perspective on suicidality restricts cultural, socio-economic, and systemic narratives about suicide and limits the questions we ask and the kinds of research we undertake. The dominance of these individualising narratives can shape approaches to intervention and support following suicide (Causer et al., 2022). Additionally, they may influence the experiences of shame, stigma and silencing that are reported by suicidal people and people impacted by the suicide of someone close to them (Hanschmidt et al., 2016).

### 1.1 Nurse suicide

Nursing is a female dominated profession globally, employing a significant proportion of women from the global ethnic majority (Kurup et al., 2024). In the UK, 89% of nurses are women, and 32.5% of all professionals registered with the Nursing and Midwifery Council are from global majority backgrounds (NMC, 2025a; NMC, 2025b). In the UK between 2011 and 2021, 398 female nurses died by suicide (NCISH, 2024). The rate of suicide for female nurses in the UK has been reported as 23% higher than for women in 367 other occupations (Office for National Statistics, 2017). More recent data reports the numbers of women nurses who have died by suicide as 28 (2020), 40 (2021) and 38 (2022), however the standardised mortality ratio for these years is not part of the data set released by the Office for National Statistics (2024), so we are unable to clarify if the rate of death by suicide among women nurses remains higher than women in other occupations. The Office for National Statistics (2017, 2024) data reports suicide by age, gender and occupation, but does not provide a breakdown of suicide by ethnicity. Therefore, it remains unknown whether the intersection of race with the experience of being a woman nurse heightens risk.

Most research that explores women nurse suicide focuses on individual and pathological explanatory factors Conolly et al. (n.d., under review).^1^ Suicide prevention policy in the United Kingdom has been described as, ‘individualised, pathologised and depoliticised’ (Marzetti et al., 2022, p1). Conversely, for all women who died by suicide between 2011 and 2021, the most common life event is reported to be financial problems and economic adversity (NCISH, 2024). Indeed, 21% of women nurses who died by suicide were reported to be experiencing economic adversity, opposed to 13% of women in other occupations. Likewise, a higher percentage were reported to have experienced workplace problems (14% v. 6%). The heightened rate of suicide among women nurses is also evident in other high-income countries including the US (Davis et al., 2021), Australia (Milner et al., 2016) and in the global north (Alderson et al., 2015). Data for the global south is under-reported, so we do not know if this is a global trend. The reasons for these differences have been attributed to pre-existing psychiatric disorders, substance use, physical health problems, and occupational and interpersonal difficulties (Groves, et al., 2023). The NCISH (2024) report suggests that ‘findings may reflect job-related features that contribute to increased suicide risk in [women nurses]’ (p7). Evidence suggests that workplace factors contribute to psychological distress across the nursing community and include high workload compounded by low resources, poor work–life balance and workplace conflict and incivility (Maben et al., 2022). In the UK, there is currently no legal requirement for workplace suicides to be investigated by the HSE and therefore organisations are not subjugated to a similar level of scrutiny and accountability in comparison to a physical injury (Waters and Palmer, 2022).

### 1.2 Suicide policy

Suicide prevention strategies are set out in national policy documents, illustrating a governmental commitment to ‘take action to prevent suicide, ideally through a comprehensive national suicide prevention strategy’ (WHO, 2021, p12). Global targets have been set to reduce the rates of death by suicide by one third by 2030; and by 15% by 2023 (WHO, 2021). In the UK this task falls to the governments of the four nations: The UK Government for England, The Scottish Parliament, The Welsh Assembly and The Northern Ireland Executive. Each has published a policy or strategy document with the aim of preventing and reducing deaths by suicide in their respective nations. Despite global targets, and these national policy strategies, the suicide rates in the UK are not falling (Department of Health and Social Care, 2023). There has been no statistically significant change in suicide rates in England between 2015 and 2021 (Garratt et al., 2023). National governments continue to set their commitment, for instance, in England, to ‘reduce the rate of suicide over the next 5 years – with initial reductions observed within half this time or sooner’ (Department of Health and Social Care, 2023). Likewise, in Northern Ireland, an aim was set to reduce the suicide rate by 10% by 2024 (Northern Ireland Executive, Department of Health, 2019).

Policy holds a unique position in setting agendas and recommending approaches toward overcoming health and social challenges attributable to broad economic, cultural, political, environmental and historic determinants. In the case of suicide prevention, current strategies have been criticised for adopting a solely psycho-centric approach (Rimke, 2016), positioning psychiatric or mental illness as being the cause of suicide (Marsh, 2016), and focusing on individual risk factors (White, 2017). More holistic approaches might consider cultural scripts (Kral, 2019), societal regulation (Abrutyn and Mueller, 2018) and interdisciplinary, collaborative perspectives to consider socio-economic and political inequalities (Mills, 2018; Chandler, 2020). Specifically in relation to women nurses, consideration of gendered approaches to suicide prevention, which is seen, statistically, as a male problem (Canetto, 2008) would highlight the needs of women who have higher rates of suicide attempt and self-harm (Fullagar and O'Brien, 2015).

There is an opportunity to consider cultural, socio-economic, gendered, intersectional and political contexts as both causative and the source for solutions when setting out policy and strategy to reduce national suicide rates. However, the strategy for achieving a reduction in the rates of suicide across the UK takes the form of focusing on ‘high risk groups’ (HM Government, 2012) that is, individuals who are identified as having a higher risk of dying by suicide due to a particular personal trait or circumstance in their life. Additionally, policy focuses on the occurrence of mental health difficulties in specific groups or individuals, illustrating the assumed relationship between mental ill-health as a pre-cursor to suicide (Gibbons, 2025); access to the means by which people might take their lives; provision of help-seeking information for individuals and for agencies who provide support; and reducing rates of self-harm (Department of Health and Social Care, 2023; COSLA: The Scottish Government and the Convention of Scottish Local Authorities, 2022; Northern Ireland Executive, Department of Health, 2019; Welsh Government, 2025). Current UK suicide prevention policy fails to explore routes toward making lives feel more liveable (Marzetti et al., 2022).

There appears to be a disconnect between the workplace experiences of women nurses, and the focus of policy documents that turn away from contextual issues, including workplaces, toward individual traits and mental health symptoms. Situating our critique theoretically, UK suicide policy’s pivot to individuated “risk” and personal coping aligns with a neoliberal rationality that reframes citizens as self-managing, enterprising subjects whose health and wellbeing are moral responsibilities rather than collective, structural concerns. In Foucauldian terms, this is an instance of governmentality: governing at a distance by inciting self-surveillance and self-regulation through expert discourse and risk metrics, what Foucault called technologies of the self. Within women’s health, Riley et al. (2019) show how “postfeminist healthism” blends neoliberal citizenship with a cultural sensibility that celebrates individual choice and consumption, translating structural inequities into personal projects of optimisation. Read through this lens, suicide prevention’s risk lists and self-care scripts responsibilise nurses and obscure the organisational, economic and gendered conditions of their work. In Foucauldian terms, the strategies we analyse operate as governmental rationalities that render suicide prevention a matter of self-conduct, shifting locus of action from states and organisations to individual nurses. Considering this, we aim to apply a critical policy analysis to national and organisational policy documents that pertain to women nurses in the UK. We aim to explore how distress, suicidality and suicide prevention in women nurses is positioned and constructed in policy and with what political, social and personal consequences?

## 2 Methods

### 2.1 Study design

We undertook a critical policy analysis of suicide prevention policies pertaining to nurses, and health workers in general, in the UK. We called on Bacchi’s (2009, 2017) ‘What’s the problem represented to be?’(WPR) method of critical policy analysis to guide the data analysis in this review. We employed this approach as it challenges the conventional view that policies are responses to problems that are situated beyond policy documents. The WPR approach asserts that it is the policy documents that contain implicit representations of the ‘problems’ that they set out to address (Bacchi, 2009), as such, WPR takes a ‘problem-questioning’ approach (Riemann, 2023). Therefore, the WPR approach allowed us to treat these problem representations as problematisations that require critical scrutiny; to ‘challenge and disrupt normalising discourses’ (Tawell and McCluskey, 2022); and to explore the effects of these representations (Bacchi, 2009).

As our population of interest were women nurses, we sought to draw out and critique the use and positioning of gender and intersectionality across the included policy documents. We drew on the concept and definition of policies as ‘gendering practices’. As Bacchi (2017, p20) sets out, ‘The suggestion, therefore, is that when we develop or analyse a policy, we ought to ask specifically how it is potentially gendering and how it may encourage the production of behaviours and characteristics conventionally associated with those called “women” and “men,” making them come to be’ (Bacchi, 2017, p20). In this definition, ‘gendering’ is described as an active process, as such we approached the policy documents with the Foucauldian conceptualisation of policy as discourse, in mind (Bacchi, 2009), and sought to identify how policy actively situates gender within process and narrative. We also engaged with Lazar’s (2007) five key principles of feminist discourse praxis, developed for application in discourse analysis, to aid in our process of identifying and critiquing discourse within policy documents.

Co-production with nurses formed a central element of our methods for the review. We have a Nurse Advisory Group who are lived experience collaborators across the wider project within which this review study is situated. Members of the Nurse Advisory Group have personal or professional experience of nurse suicide, suicidality, and/or distress. They are a diverse group of nurses who have been involved in the wider project since development of the funding bid. As such, they have contributed to shaping the project, including this review study; and in developing the research questions that this study aimed to address. The research team for the review included three Nurse Advisors. Their involvement conforms to INVOLVE standards (NIHR, 2018) and we were guided by the GRIPP SF-2 guidelines for reporting on advisory group involvement (Staniszewska et al., 2017). As co-researchers and co-authors, they worked alongside the academic authors taking equal responsibility at three key points in the review; developing the analytic framework; providing analytic oversight and reviewing and editing the draft manuscript. All three aspects of co-production are detailed below. Bacchi’s WPR and feminist discourse analysis methodology explicitly foreground the integration of analysis and interpretation. Results that are developed from these analytic methods are inherently theoretical and discursive, and separating Results from Discussion would artificially splits analytic insight from the textual material through which it is produced (Bacchi, 2009; Lazar 2007). Critical policy analyses in this genre routinely present results and discussion together (e.g., Marzetti et al., 2022; Mills, 2018) In line with this convention, our discussion is integrated into the reporting of the findings under the heading, ‘Results’.

### 2.2 Search strategy

Online searches were conducted between July 2024 and April 2025 by the lead author via Overton, Google, Google Scholar, and organisation and government websites (Table 1) to source policy, strategy and guidance documents pertaining to suicide prevention for registered nurses and health workers employed in the public, private and voluntary sectors.

**Table 1:** Governmental and organisational websites included in search strategy.

Organisation type	Search site
Governmental sites	UK Government Welsh Assembly Scottish Parliament Northern Ireland Executive
NHS sites	NHS England NHS Employers NHS Confederation NHS Wales NHS Scotland Department of Health, Northern Ireland
Private health providers	Nuffield Health Circle Health Group Cleveland Clinic London
Private providers of residential health care	HC-One Four Seasons Health Care Barchester Healthcare Care UK Bupa
Nursing unions	Nursing and Midwifery Council Unison Unite the Union
Regulatory bodies	Royal College of Nursing Care Quality Council

Search terms included:

Suicide Prevention Policy AND England OR Scotland OR Wales OR Northern Ireland.

Nurse OR nurses OR healthworker OR healthcare worker.

AND

Mental health OR wellbeing OR suicide prevention.

AND

Policy OR guidance OR guidelines OR strategy OR plan.

### 2.3 Inclusion criteria

Our review objective was to understand how policy relates to women nurse suicide in the UK. We sought to develop clarity and a strong rationale around inclusion and exclusion that was easy to articulate. Our early scoping of the policy landscape included exploration of international policy related documents, however, it became clear that establishing boundaries and appropriate inclusion and exclusion criteria around a global sample would be challenging. By limiting the searches to the four nations of the UK we were able to interrogate the included documents fully and work at analytic depth with the extracted data to ensure that reported findings pertain specifically to women nurses in the UK. Therefore, our inclusion criteria were as detailed in Table 2.

**Table 2:** Inclusion and exclusion criteria.

	Inclusion	Exclusion
Type of document	Policy; strategy; plan; guidance Document in use at the time of the document searches (July 2024–April 2025)	Primary or secondary research; opinion; evaluation; Expired or replaced policy documents
Published by	Government or organisational document	Academic institution and/or academic or professional journal or other publication. Locally published documents that pertain to one sub-region or work-group within the NHS
Topic	Suicide prevention	Suicide postvention
Population	National population or Nurses & Healthcare workers	Other professional groups or other cohorts as a sub-group of the general population
Location	Within the four nations of the United Kingdom	All other countries

### 2.4 Data extraction

Lead Author extracted data from the included policy documents for two purposes:

1. To inform an overview of the nature and attributes of the included documents, the findings of which are reported in Table 3 in the findings section and in Supplementary material A.
2. To provide data for a critical analysis of the content of the policy documents. We extracted data that was judged to be useful in answering the review question. We developed a data extraction form based on Bacchi’s six questions for this purpose.

**Table 3:** Overview of included documents (abridged version).

Document title	Publication details	Target population
Suicide prevention in England: 5-year cross-sector strategy	England, by Department of Health and Social Care Published September 2023. Valid until 2028	Whole population of England
Creating hope together: Scotland’s suicide prevention strategy 2022–2032	Scotland, by The Scottish Government and The Convention of Scottish Local Authorities Published September 2022. Valid until 2032	Whole population of Scotland
Protect Life 2: a strategy for preventing suicide and self harm in Northern Ireland 2019–2024	Northern Ireland, by Northern Ireland Executive, Department of Health. Published September 2019. Extended to end in 2027	Whole population of Northern Ireland
Understanding: the suicide prevention and self-harm strategy for Wales	Wales, by Welsh Government Published April 2025. Valid until 2035	Whole population of Wales
NHS Long Term Workforce Plan	England, by NHS England Published June 2023. Valid until 2038	NHS employees in England
A healthier Wales: our workforce strategy for health and social care	Wales, by Social Care & Health Education and Improvement Wales Published October 2020. Implementation is detailed until 2030	Health and Social Care workforce in Wales
Wales nurse retention plan	Wales, by Health Education and Improvement Wales Published September 2023. No date for review or renewal of this document	Nurses employed in NHS Wales organisations
Working together to prevent suicide in the NHS workforce: a national suicide prevention toolkit for England	England, by NHS England Published September 2023. No date for review or renewal of this document	NHS workforce in England
Creating a health and wellbeing culture: elements of health and wellbeing.	England, by NHS. Published November 2021. No date for review or renewal of this document	NHS workforce in England

### 2.5 Data analysis

All co-authors including three members of our Nurse Advisory Group collaborated to inform the analytic strategy for this review. We did this by working with extracts of data from two of the policy documents to establish the questions that we wanted to ask of the data.

We developed a four-step method of coding and analysis which we applied to the extracted data. We developed coding tables and analytic frameworks in word documents that were specifically suited to the methods we employed and the data we were analysing. The four step method, including our coding process is set out below:

#### 2.5.1 Step 1

We employed Bacchi’s (2009) ‘what is the problem represented to be’ approach to policy analysis. We undertook a close and critical reading of the included policy documents using Bacchi’s (2009) six questions (Table 4) to guide coding and memo making. We developed a code table for each document analysed; conceptual codes were logged in response to the six guiding questions. We employed an iterative approach in coding based on constant comparison (Glaser and Strauss, 1967) within and between policy documents to identify similarities, differences and silences in content, themes and discourse.

**Table 4:** WPR question framework, adapted from Bacchi (2009).

Question	Goal
Q1: What’s the problem represented to be in a specific policy?	To identify implied problem representations in specific policies
Q2: What presuppositions or assumptions underlie this representation of the ‘problem’?	To identify and analyse the conceptual logics that underpin specific problem representations
Q3: How has this representation of the problem come about?	To highlight the conditions that allow a particular problem representation to take shape and to assume dominance
Q4: What is left unproblematic in this problem representation? Where are the silences? Can the ‘problem’ be thought about differently?	To reflect on and consider the issues and perspectives that are silenced in identified problem representations
Q5: What effects are produced by this representation of the ‘problem’?	To identify the effects of specific problem representations so that they can be critically assessed
Q6: How/where has this representation of the ‘problem’ been produced, disseminated and defended? How could it be questioned, disrupted and replaced?	To pay attention both to the means through which some problem representation become dominant and the possibility of challenging problem representations that are judged to be harmful

#### 2.5.2 Step 2

Bacchi’s (2017) concept of ‘gendering’ to explore the active nature of policy in creating difference according to intersectionality (gender, race, class, ability, socio-economic status). Concurrently with Step 1, codes and concepts were organised in an analytic framework thematically and developed into narratives that articulated the dominant discourses across the included documents. Throughout the analytic process we paid particular scrutiny to gendering processes within the policy documents (Bacchi, 2017).

#### 2.5.3 Step 3

Application of a feminist critical discourse lens through use of Lazar’s (2007) five key principles. We further scrutinised the responses to Bacchi’s (2009) six questions across all policy documents according to Lazar’s five key principles for feminist discourse praxis, outlined very briefly below:

1. Feminist analytic activism: to critique discourses which sustain a patriarchal social order, identify relations of power that systematically privilege men as a social group, and disadvantage, exclude, and disempower women as a social group
2. Gender as ideological structure: to interrogate how the prevailing conception of gender as a binary was active in the policy documents.
3. Complexity of gender and power relations: to examine how power and dominance are discursively produced and/or (counter-) resisted within the documents.
4. Discourse in the (de)construction of gender: to unpick the dialectical relationship between discourse and the social in policy documents.
5. Critical reflexivity as praxis: to engage with on-going critical self-reflexivity among the research team as an analytic tool and an enactment of feminist practice.

#### 2.5.4 Step 4

Our analytic praxis drew on Foucauldian concepts of discourse, power and knowledge, governmentality and technologies of the self, and thus enabled us to trace how suicide policy produces particular subjects (“at-risk workers”) and norms (“good” self-managing employees) (Arribas-Ayllon and Walkerdine, 2017). This complements our WPR approach by clarifying how responsibilising problem-representations are assembled and defended across policy texts and organisational guidance.

Both Bacchi and Goodwin (2016) and Lazar (2007) place reflexivity as integral to their methods, and this formed a central tenet of our analytic process. The lead author used a research journal to promote reflexivity and self-awareness throughout the analysis and writing processes. As a research team, we engaged in critical conversations in team meetings, co-author meetings and supervision challenging ourselves and our colleagues to draw on self-awareness to problematise potential biases and underlying knowledges that may be shaping our interpretations and analysis.

Throughout the reflexive process critical discussion of codes, theme and discourse development were ongoing which provided both scrutiny and opportunity for reflexivity and gave the decision-making process transparency and accountability. Initial codes were discussed and interrogated for conceptual rigor. Once the initial themes (which we have named narratives) were developed, all co-authors reviewed and reflected upon them, formulating questions and points for interrogation prior to in-depth discussions in two online meetings. This process generated critique which enabled further refinement and development of the narratives and added depth to the ongoing analytical process.

## 3 Results

### 3.1 Included documents

Forty-eight documents were returned by the searches, of those 39 did not meet the inclusion criteria. Nine policy documents met all the inclusion criteria. Table 3 gives a brief overview of the included documents. A full version of this table is included as Supplementary material A. The included documents consist of national governmental suicide prevention policy and NHS documents only, illustrating that many of the organisational searches that were conducted for this review did not return documents that met our inclusion criteria.

### 3.2 Narratives

Our analysis of the data developed three narratives, the third of which encompasses four distinct themes, illustrated in Table 5.

**Table 5:** Narratives and themes developed in analysis.

Narrative	Theme
Invisible nurses and silenced suicide	
People as risky	
Responsibilising the workforce	Nurses as risky
	Knowledge and means
	A workforce problem
	A workforce solution

#### 3.2.1 Invisible nurses and silenced suicide

As we will set out in these findings, a central focus of suicide policy documents is the identification of people and groups who are seen as being at greater risk of death by suicide. Although women nurses are 23% more likely to die by suicide than women in other occupations (NCISH, 2020), we found that nurses and health-workers were largely absent from the often very comprehensive lists of ‘at risk’ groups in the policy documents of three of the four nations of the UK (Department of Health and Social Care, 2023; COSLA: The Scottish Government and the Convention of Scottish Local Authorities, 2022; Welsh Government, 2025).

Five of the documents that we reviewed pertain specifically to health workers or to nurses (Table 3). Suicide is mentioned once in the NHS ‘Elements of Health and Wellbeing’ document (NHS England, 2021), and not at all in either of the workforce strategy documents (NHS England, 2023a; Social Care Wales & Health Education and Improvement Wales, 2020), nor in the nurse retention plan (Health Education and Improvement Wales, 2023). It is only the NHS suicide prevention toolkit (which pertains to NHS staff in England) that focuses on health-worker and nurse suicide. Thus, the topic is absent within organisational policy and strategy despite the known heightened risk to women nurses in the UK. This curbs opportunities to discuss and address the issue of nurse suicide within the NHS. This silence is consistent with postfeminist healthism’s tendency to normalise a ‘normal-perfection’ for women workers while obscuring structural determinants (Riley et al., 2019) and leads us to argue that invisibility here is not accidental but produced by discourse.

We were unable to source any suicide policy or strategy documents pertaining to nurses employed outside the NHS. This is particularly worrying due to the absence of nurses in national suicide prevention policy. This presents a significant gap and is a notable oversight because, of the 788,074 nurses on the Nursing and Midwifery Council register in March 2025, only 349,015 registered nurses were working in the NHS in Jan 2024 (NMC, 2025a; NHS England, 2024a). Therefore, over half of registered nurses work in organisations other than the NHS. Further, internationally trained nurses and nurses from the global majority are over-represented in non-NHS employers such as nursing homes and social care settings (Sheehy et al., 2024). The documents included in this review were not written with those nurses in mind.

#### 3.2.2 People as risky

People with particular traits, or who find themselves in particular circumstances are identified in national policy documents as being ‘priority groups’ (England), ‘vulnerable sub-populations’ or ‘targeted group’ (Northern Ireland), or as having ‘risk factors’ (England, Scotland, Northern Ireland), and are positioned as more likely to die by suicide. These ‘risky’ people vary from one policy to another, creating a level of confusion across the UK as to who it is that policy is protecting. A detailed list of the individuals, groups and traits identified across the four national policies, and the terminology used is available in Supplementary material B. The wide range of traits, groups, experiences and circumstances that deem an individual ‘risky’ include life stages, gender, sexuality, ethnicity, traumatising events, childhood experiences, life experiences, employment status, financial status, mental wellbeing, physical health and emotional states. Some people may have multiple traits or circumstances, yet intersectionality is not acknowledged across these documents, which primarily focus on the higher suicide risk for men, neglecting women and non-binary individuals. The concept of risk within these documents locates the cause of suicide as belonging to the person, rather than being associated with external factors (Marsh, 2010). This ignores wider contexts that may contribute to suicidality including discrimination, for instance, systemic prejudice or sexism, racism, homophobia, which shape social and personal experience.

The identification and subsequent targeting of groups of ‘risky people’ can be stigmatising and lead to processes of ‘othering’ (Goffman, 1986; Scambler, 2018). This is starkly apparent when the Northern Ireland policy distinguishes between ‘population-wide’ and ‘targeted’ interventions, creating a binary between those who are seen as problematic and those who are not (Northern Ireland Executive, Department of Health, 2019). The Scotland policy illustrates how it is possible to avoid ‘risk language’ for instance, in setting out ‘our priorities’ (p13), the word ‘risk’ is notably absent. The policy text discusses the identified causes of suicide, preventative measures, identified strategies and provision of support, demonstrating that the word ‘risk’ can be taken out of the conversation, while meaning and intention remain clear (COSLA: The Scottish Government and the Convention of Scottish Local Authorities, 2022). It might be argued that the listing of ‘high risk groups’ and ‘risk factors’ raises awareness and generates knowledge, improving the likelihood of our being able to predict suicidality. However, this idea has not been evidenced over past decades as our ability to predict suicide has not improved (Large, 2018; Hawton and Pirkis, 2024). The idea of reliable prediction in suicide prevention oversimplifies ‘a complex, deeply personal phenomenon shaped by cultural and social forces’ (Gibbons, 2025, p3).

The enumeration of “risk factors” functions as a neoliberal moral economy of health, in which statistical comparators and risk profiling invite self-monitoring and self-correction by individuals rather than redistributive change by institutions. Riley et al. (2019) describe how, in women’s health, such logics are amplified by neoliberal postfeminist healthism, a cultural sensibility that equates being a “good” woman/worker with constant self-improvement and healthy consumption. In policy, this framing legitimates targeting “risky people” while silencing structural issues (gendered hierarchies, racism, precarity), thereby depoliticising suicide and shifting accountability onto those already burdened by inequity (Marsh, 2020).

The policy documents call on statistical evidence to underpin the conceptualisation of people as risky. This is mainly data derived from the UK Office for National Statistics, whose data collection largely focuses on individual risk factors. Statistics are used throughout the policy documents to establish the ‘truths’ that underpin the strategies, approaches, and action plans set out in the documents. Statistics answer the question ‘what is happening’, but not, ‘why is it happening’? The use of statistics that illustrate who is dying by suicide compared to whom else, will prompt an explanatory framework focusing on individual factors, reinforcing the idea of problematic ‘risky’ people. Exceptions to the reliance on statistical data as representing the whole truth include Scotland’s policy where the inclusion of lived experience, academic, and youth advisory boards introduced the qualitative narratives required to answer the ‘why’ questions (COSLA: The Scottish Government and the Convention of Scottish Local Authorities, 2022). The outcome of this methodological decision is reflected in the policy’s focus on socio-economic contexts such as social deprivation and poverty, debt and trauma, and the impact that they have on suicide ‘risk’. This idea is presented more explicitly than in other national policies, in acknowledging that suicide is not purely a pathological condition, and that social factors can cause harm to individuals. However, this idea could have been expanded further to include the experiences of ethnically minoritised groups, sexuality and gender identities, and the impact of health inequalities. Thus, for some, ‘risk’ remains situated in their person.

Health inequalities are acknowledged in the Northern Ireland document as being relevant to suicidality (Northern Ireland Executive, Department of Health, 2019), in connection with socio-economic inequalities. Indeed, suicide itself is recognised as a marker of inequality in this document:

‘Suicide and self-harm affect all ages and walks of life but are also amongst the starkest indicators of inequality here. Suicide rates in our most deprived areas are three times higher than in the least deprived areas’ (Northern Ireland Executive, Department of Health, 2019, p3).

Whilst marking a departure from the pervasive individualisation of suicide risk, the focus on inequalities is limited to socio-economic or health inequalities. The opportunity to explore inequality in other socially, culturally and systemically rooted experiences is missed.

The language of risk is echoed in health policy documents. For instance, the NHS suicide prevention toolkit, that pertains to all health-workers in England, lists ‘factors which may increase the risk that someone may die from suicide’ (NHS England, 2023b, p9). These include gender, age, bereavement, sexual orientation and gender identity, mental illness and social deprivation. It is notable that all but one of these factors are individual traits or circumstances, thereby positioning the heightened risk of suicide among health workers as within the individual members of the workforce. However, a final sentence in this short section of the toolkit lists a few workplace factors that may cause pressure on staff, such as burnout, moral injury and complaints. Within this section more attention is paid to ‘risk factors’ that pertain to individual workers, than to those that are associated with the workplace. This document, however, does make a rare mention of intersectional inequalities as potentially increasing exposure to risk factors, for instance, there is mention of the disproportionate number of ethnic minority staff who are subject to disciplinary processes, but little exploration of why this might be.

#### 3.2.3 Responsibilising the workforce

Within the health policy documents, our analysis uncovered a dominant narrative of workforce responsibility for the problem of, and solutions to, women nurse (and health-worker) suicide. This narrative was embedded throughout the documents within four key messages. First, nurses are positioned as being a ‘risky’ group of workers, whose heightened rate of suicide is attributed to their behaviours within their job roles. Second, nurses and health-workers have access to the knowledge and means to take their own lives, thus explaining the higher rate of death by suicide among these groups. Third, a dominant narrative of ‘workforce’ throughout the documents obscures and silences factors relating to the ‘workplace’ which is the location of the practices, cultures, learned behaviours, and environments which shape the day-to-day experience of being a nurse. Finally, the workforce is seen as the source of solutions to the ‘problem’, through responsibilities to identify colleagues at risk, to respond to identification and prevent the suicide of their colleagues. Each of these factors are explored below in more detail.

##### 3.2.3.1 Nurses as risky

Women nurses are mentioned once in the NHS England suicide prevention toolkit, in a section headed, ‘Gender’: ‘female nurses have a 23% higher rate of suicide than other women’, (NHS England, 2023b, p9) yet, despite the presentation of this startling fact, there is no further attention paid specifically to women nurses throughout the document. There is a small section that focuses on nurses as a whole group with the higher suicide rate explained as being due to a combination of factors including:

‘mounting pressures to work extended and extra shifts, self-imposed psychological pressures due to patient dependence and lack of engagement of mental health support due to fears of job insecurity or perceived collegial failure’ (NHS England, 2023b, p6).

Causation is suggested as three factors, firstly, extra and extended shifts. The use of the term ‘mounting pressure’ suggests an expectation that nurses ‘step up’ to fill vacant shifts. As such, a systemic issue becomes a moral responsibility for individual nurses to resolve. Secondly, ‘psychological pressures’ linked to ‘patient dependence’. It is not clear whether this is a judgement on the level of patient need and nurses’ ability to respond. However, there is a further judgement in the term ‘self-imposed’, thus we understand ‘the problem’ as the nurse’s response to patient dependence, with any attribution to occupational factors obscured. An alternative reading might speak of the emotional labour that nurses typically employ to meet patient demands (Hochschild, 2003; Riley and Weiss, 2016). Thirdly, the nurse’s ‘lack of engagement’ with Mental Health support. Two reasons are stated, firstly ‘fears of job insecurity’, identifying the problem as being the nurse’s fear; secondly, ‘collegial failure’ is described as ‘perceived’, again responsibilising the nurse. We could alternately recognise the psychological pressures of patient dependence as being a rational response to a difficult situation; we could position a lack of engagement, job insecurity and collegial failure all as a systemic, procedural, economic, political or cultural issues operating within teams, across hierarchies and beyond the NHS. In re-positioning these factors, we shift the focus away from the individual nurse, toward the spheres of organisational culture, economics and politics, that is, the contexts in which the nurse is working.

The document also examines the ‘risks’ that underlie female doctors’ increased rates of suicide:

‘being part of the healthcare workforce means exposure to factors that may increase the risk of suicide. This includes frequent and close proximity to traumatic experiences, and work environments and cultures associated with high levels of stress’ (NHS England, 2023b, p6-7).

There is a stark contrast in the language used, the phrase, ‘exposure to factors’, immediately locates the ‘risk’ outside the individual practitioner. The moralising descriptors used for nurses; ‘self-imposed’, ‘fear’, ‘lack of engagement’, ‘perceived’, are absent in for doctors. The doctor is positioned in ‘close proximity’ to a traumatising and highly stressful work environment, which is presented as a reasonable explanation for increased rates of suicide. The nurse, however, who works in the same environment, is positioned as being a ‘risky’ person, due to poor coping strategies and an inability to self-regulate and self-care. The differential positioning of doctors and nurses has been documented over the decades. Stein (1967), coined the phrase, ‘the doctor-nurse game’, whereby nurses have lower status and are expected to act deferentially to doctors (Essex et al., 2023). This can affect communication including the ability to speak up and can have ‘substantial person costs’ (Essex et al., 2023, p1), on the wellbeing of practitioners in less powerful positions. In the case of the NHS Suicide Prevention Toolkit, it is evident in the tones of language that are applied to nurses compared to doctors.

The differential moral tone applied to nurses versus doctors is consistent with gendered power relations with nurses positioned as lacking self-discipline, whereas doctors are depicted as exposed to external stressors. Foucauldian analysis highlights how power operates productively, not only constraining, but actively producing subjects who internalise organisational expectations (e.g., invulnerability, perfection, composure). Riley et al. (2019) note that neoliberal postfeminist sensibility normalises a “normal-perfection” for women that is unattainable yet demanded, intensifying self-surveillance and blame when distress surfaces. In this way, policy discourse codes feminine care work as personal failure rather than institutional responsibility.

##### 3.2.3.2 Knowledge and means

By way of explaining the higher rate of suicide among women nurses and health-workers, NHS England attribute this to:

‘having easy access to lethal drugs … High risk of suicide among health professionals could also be explained by these occupations possessing relevant knowledge on methods of suicide’ (NHS England, 2023b, p6).

Health workers are more likely to use poisoning as a means of suicide than the general population. For instance, 43% of women nurses who die by suicide use self-poisoning compared to 29% of women in the general population (NCISH, 2024). This statistic underpins the statement made above in the NHS suicide prevention guidance (NHS England, 2023b). It is important however, to explore the context of the statistic, as such, in the case of women nurses who were also mental health patients:

‘the main drugs taken by female nurse patients were psychotropics (26%), opiates (21%), and paracetamol (13%). For female patients who used opiates in self-poisoning, nurses were 5 times more likely to have been prescribed these opiates than women in other occupations (8, 62% v. 32, 24%).’ (NCISH, 2024, p6).

A contradiction exists between the use of prescribed medication for this purpose and the statement in the NHS England guidance, that health-workers have ‘easy access to lethal drugs’, inferring that drugs are obtained unethically and illegally from the workplace for the means of suicidal activity. However, the unauthorised procurement of hospital medication by staff for personal use would not be a simple process (CQC, 2022). The suicidal nurse, rather than receiving support at a time of distress and personal crisis, is framed as a ‘risk’ to security processes.

Framing women nurses’ suicides as a consequence of specialised knowledge or means is a classic individualising move that obscures how workplace regimes (staffing, disciplinary exposure, moral injury) and biopolitical governance shape vulnerability. A Foucauldian perspective would treat “means” not as a personal moral deficit but as an artefact of institutional arrangements, while postfeminist healthism explains how women workers are incited to privatise distress and manage it silently to remain the “good” professional.

The use of statistics to underpin this narrative is problematic for a number of reasons. It excludes a qualitative narrative, and any exploration of why this is a problem; exploration of the underlying issues that lead to suicidality is absent; health-workers who die by other means of suicide are also absent from this construction. Focusing on the behaviours of individuals who are already suicidal ignores the contextual factors that may contribute toward suicidality. Thus, opportunities for understanding to inform solutions are missing. Finally, this explanation situates the problem within members of the workforce, ignoring the workplace.

##### 3.2.3.3 A workforce problem

The NHS Suicide prevention toolkit is entitled, ‘working together to prevent suicide in the NHS workforce’ (NHS England, 2023b, title page), and aims to:

‘assist organisations to embed suicide prevention strategies in the organisation’s health and wellbeing policies and guide the approach to supporting those at risk of suicide within the workforce’ (NHS England, 2023b, p3).

By constructing nurse suicide as a ‘workforce’ rather than a ‘workplace’ problem, attention is directed toward individual nurses rather than toward work environments, practices, cultures and systems. This presents two problems. First, the workforce is seen as a ‘homogenous whole’, rather than as the most diverse it has ever been (NHS England, 2025). Therefore, multiple contexts relating to social, cultural, historic and economic experiences are overlooked; as are workplace experiences such as bullying and violence, and power imbalances. Second, as workplace issues are unidentified and unaddressed, individual staff who die by suicide are seen as wholly responsible for their own deaths, which are constructed as unconnected to the workplace context (unless the means of death were illicitly obtained drugs, as mentioned above). The opportunity to understand the abundant and nuanced experiences that exist within the workforce, and their relation to suicidality, is missed.

Two related concepts are present in the NHS documents that focus attention on the individual worker and are presented as potential solutions to not only suicide, but also other issues, such as staff retention and staff sickness. They are ‘a mentally healthy workforce’ and ‘improving personal health and wellbeing’. A ‘mentally healthy workforce’ (rather than workplace) is introduced in the NHS suicide prevention toolkit (NHS England, 2023b), as a strategy for suicide prevention. The concept is defined as:

‘the fostering of a positive culture by encouraging healthy and supportive behaviours such as thanking people for what they do, recognising and valuing their work colleagues deliver, supporting healthy eating, hydration and access to fresh air, being kind to each other, noticing when a colleague is out of sorts and asking them about it and providing a healthy work environment which promotes team away days and team development to strengthen team relationships’ (NHS England, 2023b).

Read through Foucauldian ideas regarding governmentality, “healthy culture” checklists operate as soft governance which effectively means codifying “what good looks like” so workers govern themselves and each other (peer surveillance; “noticing” colleagues) (Riley et al., 2019). Riley et al. (2019) have shown how self-care is repackaged as moral duty within postfeminist healthism, re-legitimising scrutiny of women’s bodies and emotions under the banner of health and exporting organisational burdens onto individual workers. This subtly recasts structural harm as personal compliance, leaving workload, hierarchy and discrimination untouched.

The ‘fostering of a positive culture’ sounds like a good strategy in supporting employee wellbeing. However, the focus is on individual behaviours and actions and looking after the wellbeing of each other. Responsibility is not clarified so we do not know if some of these instructions are for employers or whether staff are being instructed in self-care. The paternalistic promotion of behaviours such as saying thank you and being kind as a route toward improving staff wellbeing feels somewhat simplistic and very patronising given that care-work is a gendered and undervalued profession, currently operating in an organisation that is under-funded, under-staffed, and operating with a workforce that continues to experience the impact of trauma following the pandemic (Maben et al., 2022; Conolly et al., 2024). The suggestion of team away days and development within a ‘healthy work environment’ also feels at odds with the current workforce and resource crises within the organisation. The desired outcome is stated as being a strengthening of team relationships, however there is no evidence is provided to underpin the assumption that stronger team relationships prevent suicide.

The NHS ‘Elements of health and wellbeing’ document (NHS England, 2021) focuses on the concept of ‘Improving personal health and wellbeing’ (pp 6–19) as one of seven strategies that together will create a ‘healthy workplace culture’. Positioning this as the first section in the document suggests it is viewed as a priority by the authors, and again responsibilises the workforce with the task of generating change in the workplace. Thirteen pages focus on educating the health workforce in how to stay physically and mentally healthy. This section has twice as much ‘page space’ as any of the other six sections, including those on ‘the workplace environment’ and the ‘role of managers and leaders’. Subheads in this and other sections include: ‘Healthy lifestyle’; ‘Bringing your whole self to work’; ‘Supporting each other’; and ‘Life balance’. Two themes run through the document, firstly, a focus on the positive and important impact that following the suggested actions will have on colleagues and patients. Secondly, a repeated theme of ‘what does good look like’, where expectations for optimum behaviours are set out for staff. Thus, the document centres on the premise that individual staff members are responsible for changing their behaviour and practice in prescriptive ways to improve the experiences of others.

##### 3.2.3.4 A workforce solution

Just as the ‘problem’ of nurse suicide is positioned within the workforce, so is potential solution. The NHS Suicide prevention toolkit (NHS England, 2023b) asserts that staff be made aware of those people who are at higher risk of dying by suicide and have awareness of the ‘signs’ of suicidal ideation or behaviour, to be ready to provide support or crisis intervention to their colleagues. There is no debate in the document about whether colleagues are the best placed people to raise concerns with each other about suspected suicidal behaviour, or warning signs. In fact, training for staff members is suggested (although not compulsory) via a Zero Suicide online 20-min video training (Zero Suicide Alliance, 2024) which is assumed to give staff members the skills needed to have conversations about suicidality with each other. In this way individuals within workforces can be viewed as social actors in their own policing with organisational policies dictating behavioural norms to create individual compliance in these acts of surveillance. This is reminiscent of Foucault’s ideas regarding technologies of power which govern human conduct at a distance and are frequently embodied in the form of technologies of the self in which human beings seek to regulate their conduct (Arribas-Ayllon and Walkerdine, 2017: 118).

A list of ‘signs’ that a person may be struggling (NHS England, 2023b) is organised by ‘changes in productivity; changes in social functioning; changes in personality or behaviour’ (p9). Thus, the onus is on the ‘struggling’ individual to demonstrate their vulnerability within a culture that prizes invulnerability and legitimises organisational rules regarding emotions (or ‘feeling rules’, Hochschild, 2003; Kirk et al., 2021). The responsibility is also on colleagues to notice these changes and to initiate a conversation exploring them. Reliance on a list of ‘signs’ as a preventative tool is ‘risky’ for two reasons. First, nurses are operating in a ‘patient first’ culture and are noted for their tendency to put the care of others before their own needs. It is likely, with these deeply valued professional traits (Jackson et al., 2021) that nurses will in fact hide any signs of personal struggle to comply with the expectations of their role in a work environment that busy, stressful and under-resourced (Kinman and Leggetter, 2016). In fact, a key element of nursing, intense contact with patients and delivery of compassionate care, has been shown to involve emotional labour, the performing of managed emotions to ease the fear and distress of others (patients), whilst hiding their own emotions (Hochschild, 2003). This privatisation of feelings is a form of self-regulation; possibly a response to the expectation that nurses should be ‘invulnerable’, or to the stigma of expressing emotion in the workplace. The outcome is that distress remains unseen, leading to a lack of recognition and support for the emotional and physical demands of the job. Secondly, placing the onus on colleagues to be able to observe signs of suicidality in their colleagues within a fast-paced and stressful work environment feels unachievable. We question whether nurses have the time and capacity to monitor and respond to the wellbeing needs of their colleagues? Significant silences in this narrative include debates around the emotional burden of listening to the wellbeing struggles of colleagues; the ethical dilemmas that might be presented about how to respond, who to tell, and how to keep their colleague safe; the moral distress that might be experienced if the response to a raised concern does not align with the staff members hopes or beliefs about best next steps; the conflicts of interest between giving attention or energy to the burdens of colleagues when there are patients who need time and attention; the power imbalances that leave nurses feeling unable to raise concerns about anything within work places (Jones and Kelly, 2014); or, simply, the stigma associated with, and the skill and delicacy needed when, talking about suicide.

As noted, certain groups within the workforce are identified (or labelled) as ‘risky’ in terms of suicidality. It is possible that individual staff may be singled out as ‘at risk of suicide’ and become subject of surveillance (and gossip) within their team. Such instances may allow established prejudices to be validated, or may lead to scapegoating, bullying, stigma, discrimination, racism and the denial of development and career opportunities. In responsibilising staff for their colleagues’ wellbeing, they may feel burdened with the responsibility of any staff suicides that do occur. The outcome of a colleague’s death by suicide in the NHS can include a sense of having ‘missed the signs’, of having let their colleague down, intense guilt and failure, and complex anger (Conolly et al., 2024).

The concept that suicide is everybody’s business runs through the included documents. The ‘everybody’s business’ narrative is prevalent in national suicide prevention policy, and the messaging of suicide prevention and suicide awareness organisations (Samaritans, 2025). The England suicide prevention strategy states:

‘there is more we can all do to ensure we are all equipped with the skills necessary to potentially save lives’ (Department of Health and Social Care, 2023).

This concept is underpinned by a number of strategies, including that everyone should have access to training and support; that there is ‘no wrong door’, a concept that proposes no matter who you turn to for support, that you will receive support (NHS England, 2024b); provision of workplace mental health and wellbeing support; and the idea of a ‘national conversation’, ‘using appropriate language, to nurture a sense of responsibility, to reduce shame and stigma’ (Department of Health and Social Care, 2023, p64). The assumption is that individuals and organisations could affect, through behaviours, language and policy, a reduction in the rates of suicide.

Training staff to recognise “signs” and initiate peer interventions exemplifies technologies of the self (Arribas-Ayllon and Walkerdine, 2017) embedded within participatory surveillance with colleagues enlisted to monitor productivity, affect and comportment as indicators of risk. This reproduces a culture where visibility and confession are prerequisites for care, conditions women are culturally discouraged from meeting (Riley et al., 2019).

In Scotland the concept of ‘everybody’s business’ is again employed (COSLA: The Scottish Government and the Convention of Scottish Local Authorities, 2022). Here the ‘everybody’ refers to partners in the NHS, social care, public health, criminal justice and education. The vision includes promotion of ‘awareness, understanding, knowledge and skills’ (COSLA: The Scottish Government and the Convention of Scottish Local Authorities, 2022, p27) in order to enable these workers to be proactive in suicide prevention. What is unsaid is that these groups of professionals are already carrying the burden for delivering their own remit in sectors that are underfunded and understaffed. Additionally, these are the same workers who are tasked with identifying other sets of signs and symptoms that are also ‘everybody’s business’, for instance, in safeguarding (Local Government Association, 2023). These professionals become overburdened with not only their own remit but carrying shared responsibility for everybody else’s remits too.

The notion of ‘everybody’s business’ is explicit in the NHS suicide prevention toolkit (NHS England, 2023b):

Suicide is not inevitable, and suicide prevention is everyone’s business’ (NHS England, 2023b, p5).

The strategies of employing staffs’ observations of each other, and the expectation that they will address concerns with each other are underpinned by this concept. It might be argued that this feels like a covert screening programme. An important question that goes unasked, and therefore unaddressed across all these documents is, does ‘everybody’s business’ result in suicide prevention being ‘nobody’s job’?

## 4 Conclusion

In this critical policy analysis, we set out to explore how distress, suicidality and suicide prevention in women nurses is positioned and constructed in UK policy and with what political, social and personal consequences. We adopted a critical feminist approach to interrogate the data and draw out issues pertinent to women nurses. Co-production with women nurses was central to our study design, aligning with our mission to undertake research with nurses, for nurses. Three members of our Nurse Advisory Group made meaningful and impactful contributions throughout the study, informing our shared understanding of the data. Our analysis reflects their perspectives, and they are co-authors on this paper.

We found that suicide prevention policy documents adopt a language of risk to a wide range of individuals, minoritised groups and life circumstances thus defining the ‘problem’ of suicide as being situated within individual people. Our findings therefore converge with critiques of neoliberal responsibilisation and postfeminist healthism. Policy problematisations render suicide a matter of individual deficits (resilience, coping, help-seeking) and morally charged self-care, while failing to engage biopolitical and workplace determinants (inequality, power, resources). A Foucauldian lens illuminates how these texts produce nurses as governable subjects via risk discourse and codified norms, inciting self-discipline while depoliticising organisational accountability. This approach pathologises and problematises people and aligns with the bio-medical model which is prominent in suicide research as noted by Conolly et al. (n.d., under review, see footnote 1). Reliance on the concept of risk as being central to understanding suicidality and preventing deaths by suicide detracts from systemic discrimination and socio-economic inequalities as potential causes and possible systemic and society level solutions. The included policy documents relied heavily on statistical data to frame individuals and groups as ‘risky’. This is problematic as it can be blaming and stigmatising for people who are already experiencing challenges or vulnerability. In addition, the dependence on statistics silences lived experience and meaning in the development of policy narratives and strategies, creating policy that is done to people, rather than with people.

There is, however, evidence of movement toward a context focused direction. For instance, Scotland’s engagement with advisory groups in the development of their national policy generated consideration of socio-economic factors and poverty as contributing to suicidality. There remains space however, to increase this direction of movement extensively. For instance, as we have highlighted, workplace experiences such as violence and bullying are absent in these policy documents. Current policy is largely race and gender blind, and neglects consideration of intersectionality. By stopping short in this way, policy makers stifle the potential to grow our understanding of the underlying reasons as to why some individuals and groups are more likely to die by suicide than others.

Repositioning the individual by spotlighting structures, systems and workplaces as potential contexts contributing to suicide may be a more effective approach to suicide prevention. For instance, when we talk about people as risky, we are effectively positioning them as the cause of heightened rates of suicide, and prevention measures focus on screening and surveillance of individuals to limit their opportunities to act on their suicidality. Rather, we could position suicidality – the suicidal person – as a symptom, prompting curiosity around the causes of their suicidality. This shift in perception might happen through including lived experience individuals and groups in the conversation, to explore the systemic and structural contexts which affect their lived experience – as demonstrated, in part, in the national policy for Scotland (COSLA: The Scottish Government and the Convention of Scottish Local Authorities, 2022). Thus, policy might become less about person-focused interventions to stop suicidal people from dying; and more about social, economic and political strategies and change aimed at preventing the onset of suicidality.

Regarding women nurses, we found that they are largely absent within the policy documents of the four nations of the UK, and that suicide as a topic is also absent in health services policy. Health policy also falls short in looking at social contexts, or work environments. Research illustrates the kind of workplace challenges that nurses face (Taylor et al., 2024), and workplace factors have been linked to nurse suicidality (Groves et al., 2023), yet the consistent narrative in health service policy is of the workforce, who are responsibilised for the rate of nurse suicide and with providing solutions to nurse suicide. Rather the authors of policy and strategy documents and toolkits could turn their attention to these identified workplace factors, and as with national policy, shift from legislating for symptoms of suicidality, toward legislating to combat the systemic, discriminative and culturally ingrained behaviours that shape the working lives of women nurses. Policy can be seen as a site of cultural and organisational change, adopting a perspective of innovation and opportunity it is possible that policy can be the conduit through which equitability in the workplace is established, through the distribution of power and resources.

We would like to acknowledge that this study purposively restricted the analysis of documents from the four nations of the United Kingdom, and that this may pose a limitation in translating the findings in international settings. This policy analysis sits within a larger project which focuses wholly on the experiences of women nurses in the UK, which informed our decision making. Similarly, the inclusion of nine documents in the analysis may be seen as a relatively small number. In designing the inclusion and exclusion criteria for this study, we did not know how many documents we would find. We have been able to interrogate these documents in great depth and detail, which may not have been possible with a larger number of included texts.

## 5 Recommendations

### 5.1 For all suicide prevention policy makers

- Focus on the discrimination and social and economic inequalities that make lives less liveable, rather than individual risk factors.
- Include broad and open consultation with all groups (beyond topic experts) affected by and targeted in policy documents, including marginalised groups.
- Seek to engage with qualitative and co-produced approaches as well as statistical evidence to ensure that policy recommendations are holistic.
- Scrutinise all evidence especially statistics to explore what they are reporting, about whom under which circumstances.
- Look at diversity within both the causes and the source of solutions for suicidality.
- Solutions need to be nuanced and considerate of the diversity within the focus population.
- Pay attention to language used within suicide policy documents, and how blame or responsibility may be apportioned to particular individuals or groups, and how this might in fact increase stigma around suicide.
- Shift focus from preventing suicidal behaviour through risk management toward preventing suicidality through making lives more liveable.
- Language matters. Explicitly audit policy language for governmentality effects (where responsibility and surveillance are exported to individuals), and revise to prioritise structural levers (such as staffing, equity, anti-racism, power redistribution) over behavioural compliance.
- Integrate postfeminist healthism insights when designing gender-responsive strategies (such as avoiding scripts that implicitly equate “good” woman workers with unlimited self-optimisation and silent emotional labour).

### 5.2 For health services policy makers

- Incorporate suicide prevention within documents that pertain to wellbeing, staff sickness and retention, workforce and organisational planning documents.
- Acknowledge and work to address ingrained narratives such as invulnerability and recognise the workplace as a location of trauma and seek to connect these factors with staff suicide.
- Engage with these issues as sites for providing staff support and making change to address staff suicide, issues of wellbeing, and sickness and retention.
- Use policy as a tool to establish equitable status across professions and redistribute power and resources equally across workplaces and workforces.
- Replace peer-surveillance/self-care toolkits with organisation-level interventions such as safe-staffing mandates, protected time, psychological safety to speak up across hierarchies, and equity frameworks that address race/class/gendered harms known to increase distress and suicidality among women nurses. These should be framed as employer duties rather than worker virtues.
- Re-centre gendered, intersectional and structural contexts to disrupt existing problem-representations and redirect prevention toward making lives and workplaces more liveable.

### 5.3 For future research

In light of our findings and discussion, we advocate for further research to explore the following:

- The explanatory discourses that nurse leaders, policy makers and senior stakeholders employ with regard to the heightened rate of death by suicide among women nurses
- The explanatory discourses that nurses themselves employ with regard to the heightened rate of death by suicide among women nurses
- An exploration of women nurses experiences of stress, distress and suicidality
- Exploration of public perceptions regarding nursing, nurses and suicide among women nurses
- Exploration of the experiences and views of internationally qualified nurses who are practicing in UK healthcare settings
- Exploration of the impact of complaints processes and fitness to practice procedures on women nurses
- Explorations of women nurses experiences of workplace violence and bullying and whistleblowing processes
- We advocate for the use of critical, feminist and intersectional methodologies to explore the above phenomena, firstly to counter the dominance of positivist and individualising methodologies in this topic area; secondly to ensure that women nurses experiences are contextualised within the cultural, organisational, social and political settings in which the profession of nursing is situated and the acts of nursing occur.

## Footnotes

1 Conolly, A., Causer, H., Oates, J., Shannon, C., Knight, E., Anetekhai, C., et al. (n.d.). Suicidality in women nurses: a critical interpretive synthesis (under review).
