# Supplementary material for: The positioning of women nurses as ‘risky’ in United Kingdom suicide prevention policy documents: a critical policy analysis
Source: Front Sociol. 2026 Feb 23;11:1730850. doi: 10.3389/fsoc.2026.1730850 (PMC12967938; doi:10.3389/fsoc.2026.1730850)
Supplement: Supplementary file 1 [file Table_1.docx]

Supplementary Material

#### Supplementary materials A: Overview of included documents

| **Policy name & dates** | **Publication country; publication and expiry dates** | **Authors** | **Target population** | **Methodology** | **Vision/Aims/Outcomes** | **Priorities/Themes** |
| --- | --- | --- | --- | --- | --- | --- |
| Suicide prevention in England: 5-year cross-sector strategy:  Published | England  11.09.2023  Valid until 2028 | Department of Health and Social Care.  Forwards by: Maria Caulfield MP & Prof Sir Louis Appleby | Whole population of England | Methodology is not outlined. Brief references to data, research evidence and the role of people with lived experience. Lack of clarity around sources of evidence and methods of synthesis. | - Reduce the suicide rate over the next 5 years. - Continue to provide support for people who self-harm. - Continue to provide support for people who have been bereaved by suicide. - No identified outcomes | - Improving data and evidence - Tailored, targeted support to priority groups - Addressing common risk factors at population level - Promoting online safety & responsible media content - Providing effective crisis support - Reducing access to means and methods of suicide - Providing effective bereavement support - Making suicide everybody’s business |
| Creating Hope Together: Scotland’s Suicide Prevention Strategy 2022 - 2032 | Scotland  September 2022  Valid until 2032 | The Scottish Government and the Convention of Scottish Local Authorities (COSLA).  Forward by Minister for mental Wellbeing and Social Care & the Health and Social Care Spokesperson (COSLA) | Whole population of Scotland | Methods are detailed clearly and include public engagement; an academic advisory group; a youth advisory group  **Four key sources of input are:** Lived experience; Data and Evidence; Subject expertise; Practice insight.  **Theoretical foundation:**  The strategy is informed by the IMV framework (The Integrated motivational-volitional model) | Our **vision** is to reduce the number of suicide deaths in Scotland, whilst tackling the inequalities which  contribute to suicide.  Support for our communities to become safe, compassionate, inclusive, and free of stigma.  Our **aim** is for any child, young person or adult who has thoughts of taking their own life, or are affected by suicide, to get the help they need and feel a sense of hope.  **Outcomes** include:   - Environmental conditions. - Community understanding and organisational support. - High quality, accessible support for anyone who is suicidal and for those who support them. - Collaborative delivery - between national, local and sectoral partners. Regular monitoring, - evaluation and review. | **Guiding Principles:**   - We will consider inequalities and diversity. - We will ensure our work is relevant for urban, rural, remote and island communities. - We will co-develop our work alongside people with lived, and living, experience. - We will ensure the principles of Time, Space, Compassion are central to our work. - We will ensure the voices of children and young people are central to our work. - We will provide opportunities for people across different sectors at local and national levels to - come together, learn and connect. - We will take every opportunity to reduce the stigma of suicide. - We will ensure our work is evidence informed, and continue to build the evidence base through evaluation, data and research. |
| Protect Life 2  A Strategy for Preventing Suicide and Self Harm in Northern Ireland 2019-2024 | Northern Ireland  September 2019  (Now extended to end in 2027) | Department of Health.  Forward by Permanent Secretary for Dept of Health. Richard Pengelly & Conor McCafferty M.Sc. Reg. MBACP (accred) Chair of Suicide Strategy Implementation Body | Whole population of Northern Ireland | No methodology section.  In text statements about evidence base and collaboration are not underpinned with detail of ‘what’ or ‘how’.  A consultation report (2016) is linked.  Governance, progress monitoring and evaluation are mentioned.  An evaluation framework and performance indicators are included as an appendix  **Theory:**  The Zero Suicide model is mentioned in relation to patient safety.  In text reference to  Joiner’s (2005) Interpersonal – psychological theory of suicide behaviour. | - Reduce the suicide rate in Northern Ireland by 10% by 2024. - Ensure suicide prevention services and support are delivered appropriately in deprived areas where suicide and self-harm rates are highest. | **The broader population approaches include:**   - increasing awareness of suicide prevention - reducing stigma associated with suicidal behaviour and mental illness - promoting help-seeking behaviour, especially in males - training those who interact regularly with vulnerable or at risk people so that they can pick up early signs of acute distress and mental ill health - improving data collection and analysis to inform service improvement - restricting access to the means of suicide, where possible.   **Targeted interventions focus primarily on:**   - those who self-harm and their families - those who are in emotional crisis or who are already suicidal, and their families - those who are bereaved by suicide and those who are exposed to suicidal behaviour in others - reducing suicide risk among those in contact with mental health and substance misuse services.   **Objectives:**   - Ensure a collaborative, co-ordinated cross departmental approach to suicide prevention. - Improve awareness of suicide prevention and associated services. - Enhance responsible media reporting on suicide. - Enhance community capacity to prevent and respond to suicidal behaviour within local communities. - Reduce incidence of suicide amongst people under the care of mental health services. - Restrict access to the means of suicide. - Enhance the initial response to, and care and recovery of people who are suicidal. - Enhance services for people who self-harm, particularly for those who do so repeatedly. - Ensure the provision of effective support for those who are exposed to suicide or suicidal behaviour. - Strengthen the local evidence on suicide patterns, trends and risk, and on effective interventions to prevent suicide and self-harm. |
| Understanding:  The Suicide Prevention and Self‑harm Strategy for Wales | Wales  02.04.2025  Valid until 2035 | Welsh Government  Forward by Sarah Murphy MS Minister for Mental health and early years. | Whole population of Wales | States: ‘extensive engagement with stakeholders across Wales’.  The strategy is embedded within the broader policy context of: A Healthier Wales: our Plan for Health and Social Care: a whole system approach to health and social care in Wales.  Commitment to update delivery plans as new evidence emerges.  A ‘delivery plan’ and ‘outcome framework’ will guide implementation and evaluation against identified outcomes. | Our **Vision** for Wales is:  The annual rates of suicide in Wales will continuously decrease. People who self-injure, self-poison, who have suicidal ideation or have attempted suicide will feel safe and understood. They will belong to informed and compassionate communities and will be able to access support and services which meet their needs when and where they need them.  The **objectives** within this Strategy are designed to:   - - Increase our understanding of suicide and self‑harm   - Recognise and develop the contributions of all Government departments   - Improve support for people who self‑harm, irrespective of suicidal intent.   - Improve support for family, friends or anyone impacted by self‑harm and suicide.   **Outcomes:**   - An annual reduction in the number and rates of suicide and attempted suicides. - Better outcomes for people accessing support for self‑harm and suicidal ideation. - Better outcomes for people affected by suicide and self‑harm.   An outcomes framework will be published alongside the strategy document.  A three-year delivery plan is published with detailed actions. | Delivery is organized under six strategic objectives:   - Listening and learning - Preventing - Empowering - Supporting - Equipping - Responding |
| National Health Service Long Term Workforce Plan | England  June 2023  Valid until 2038 | NHS England  Foreword by Amanda Pritchard, NHS Chief Executive | NHS employees in England | In text statement:  ‘While the Plan’s model is founded on data, evidence and analysis, its projections are made with assumptions that are, by their nature, subject to a degree of uncertainty.’  Evidence:  **Statistical data** is used to underpin the aims of the plan. **Methods** of modelling projections that have informed the plan are detailed.  **Consultation:**  An appendix lists multiple organisations ‘engaged in forming’ the plan. These include, Royal Colleges; Professional bodies; Think tanks; Trade unions; Regulators; NHS Organisations; Education; & Other organisations.  The nature of engagement is not detailed. It is not clear what, if any, consultation occurred the workforce. | There is no succinct articulation of aims, vision in this plan.  No outcomes are articulated in this plan. | **Three key themes:**  **Train:** significantly increasing education and training to record levels, as well as increasing apprenticeships and alternative routes into professional roles, to deliver more doctors and dentists, more nurses and midwives, and more of other professional groups, including new roles designed to better meet the changing needs of patients and support the ongoing transformation of care.  **Retain:** ensuring that we keep more of the staff we have within the health service by better supporting people throughout their careers, boosting the flexibilities we offer our staff to work in ways that suit them and work for patients, and continuing to improve the culture and leadership across NHS organisations.  **Reform:** improving productivity by working and training in different ways, building broader teams with flexible skills, changing education and training to deliver more staff in roles and services where they are needed most, and ensuring staff have the right skills to take advantage of new technology that frees up clinicians’ time to care, increases flexibility in deployment, and provides the care patients need more effectively and efficiently. |
| A Healthier Wales:  Our Workforce Strategy For Health And Social Care | Wales  October 2020  Implementation is detailed until 2030 | Social Care Wales & Health Education and Improvement Wales. | Health and Social Care workforce in Wales | **Engagement and Consultation:**  Engagement Jan – July 2019 = over 1000 people contributed;  Cosultation Document July – Sept 2019: 200 responses.  Draft Strategy Dec 2019.  A report by the Kings Fund is referenced in setting the case for change: [What’s Your Problem, Social Care? The Eight Key Areas For Reform \| The King's Fund](https://www.kingsfund.org.uk/insight-and-analysis/long-reads/whats-your-problem-social-care) | A**mbition**: we will have a motivated, engaged and valued, health and social care workforce, with the capacity, competence and confidence to meet the needs of the people of Wales. Specifically, this means that;  • We will have a workforce with the right values, behaviours, knowledge, skills and confidence to deliver evidence based care, and support people’s wellbeing as close to home as possible.  • We will have a workforce in sufficient numbers to be able to deliver responsive health and social care that meets the needs of the people of Wales.  • We will have a workforce that is reflective of the population’s diversity, Welsh language and cultural identity.  • We will have a workforce that feels valued and is valued.  **Outcomes** are identified under each theme within the strategy. | Seven themes:   - An Engaged, Motivated and Healthy Workforce - Attraction and Recruitment - Seamless Workforce Models - Building a Digitally Ready Workforce - Excellent Education and Learning - Leadership and Succession - Workforce Supply and Shape   Three fundamental principles:   - Wellbeing - Welsh Language - Inclusion |
| Wales Nurse Retention Plan | Wales | NHS Wales: Health Education and Improvement Wales (HEIW) | Nurses employed in NHS Wales organisations | Details of a **literature review** cites evidence from: Professional Bodies and think tanks (The Kings Fund, Nuffield Trust, and Advisory Board).  **Data** from the NMC 2022 survey underpins the plan. | The Plan is aimed at supporting the retention of nurses employed in NHS Wales organisations. It is suggested that the plan be adopted by other employers across the health and care system.  **Evaluating Impact**  Organisations are tasked with evaluating the work undertaken on actions to improve nurse retention. Brief guidance is detailed.  **What will be different:**  • Our workforce feels valued, is treated fairly and their wellbeing is supported  • Worforce language, culture and diversity reflects our population  • Potential shortage areas are known earlier and targeted effectively  • Widespread values based and inclusive recruitment ensures we have the right people  • Common competences are identified and underpin new and diffferent ways of working  • Learning is delivered through flexible and accessible routes  • Widespread digital skills capability underpins care delivery  • National bi-lingual careers service is widening access to careers in health and care for all ages.  **What will success look like:**  • Very high levels of staff engagement, motivation, wellbeing and satisfaction  • Better recruitment and retention of staff through attractive and flexible working arrangements and career opportunities  • Increased levels of Welsh language skills in the health and care workforce  • Flexible education opportunities and career development  • Intelligence led workforce planning enabling us to change our workforce to meet our population need  • A compassionate culture, role modelled by excellent leaders and managers. | An **action plan** is presented under the following headings:   - Compssionate leadership and culture - Valuing our staff - Supporting new starters and those changing roles - Supporting international nurses - Development and career planning - flexible working - health and wellbeing - recognition and incentives - Evaluating impact - Understanding data and information |
| Working together to prevent suicide in the NHS workforce: A national suicide prevention toolkit for England | England, Sept 2023  No date for review or renewal of this document | NHS England,  Foreword by Dame Ruth May, chief nursing officer, NHS England. | NHS workforce in England | No methodology section. Some sources are cited for statistical data that evidence ‘the problem’. | To provide an organisation level toolkit to raise awareness of poor mental health and suicidality among healthcare staff  across all healthcare settings.  • Provide information enabling all staff to be able to signpost  healthcare colleagues to mental health and wellbeing.  • Provide examples of good organisational practice in  preventing suicide in staff working in healthcare.  • Provide recommendations to support organisations’ suicide  prevention strategies.  No **outcomes** are identified in this document | Three sections set out the strategy:   - Key strategies for suicide prevention - How to identify healthcare staff at risk of suicide - How to respond to warning signs and support healthcare staff. |
| Creating a health and wellbeing culture: Elements of health and wellbeing. | England.  No publication or review/renewel dates on the document. | NHS – Not stated. | NHS workforce in England | No account of methods employed to inform this strategy document. | This resource aims to provide the evidence base and inspiration for change for all [the] components of health and wellbeing.  There are sections titled **‘what does good look like’** throughout the document.  No identified set of **outcomes** in this document. | Illustrating the case for health and wellbeing in the NHS.  Including:   - defining health and wellbeing for the NHS across organisations - academic evidence supporting health and wellbeing interventions - business case supporting data for NHS health and wellbeing from across the NHS and the broader UK workforce - things to consider for your organisation when approaching health and wellbeing |

#### Supplementary materials B: Individual traits, groups and circumstances identified as associated with heightened rates of death by suicide, and the descriptors used in the policy documents of the four nations of the United Kingdom

| **England**  Risk factors | **England**  Priority Groups | **Scotland**  Risk factors | **N. Ireland**  Risk factors | **N. Ireland**  Vulnerable Sub-Populations | **N. Ireland**  Targeted Groups | **Wales**  An all-age approach |
| --- | --- | --- | --- | --- | --- | --- |
| Physical Illness  Financial difficulty & economic adversity  Harmful Gambling  Substance Misuse  Domestic Abuse  Going through divorce or separation  Social Isolation | Children & Young People  Middle aged Men  People who have self-harmed  People in contact with Mental Health services  People in contact with the justice system  Autistic People  Pregnant women and new mothers | Age and sex should be considered when other risk factors of suicide attempt are present, for example, self-harm history, impulsivity, and feeling of entrapment.  Specific risk factors include: employment status, a sense of defeat, hopelessness, and  Challenging relationships.  Perceptions of relationships should be considered, for example, feelings of burdening others.  Intergenerational interventions can help avoid negative experiences in early life, for example, poor maternal mental health in the antenatal period is associated with negative impact on the emotional, physical and mental development of the child, during infancy and the early years, but also through to teenage years.  Interpersonal connections should be developed / improved to help maintain relationships (as they provide vital social support). | **Individual Level**  Previous suicide attempt  Family history of suicide  Chronic pain, debilitating physical illness  Physical and sensory disability  Mental disorders  Alcohol and substance misuse  Hopelessness  Unemployment, job or financial loss, unmanageable debt, poverty  **Relationship Level**  Sense of isolation and lack of social supports  Relationship conflict, discord or loss  Unstable / unsupportive parent – child relationships  Bereavement  **Community Level**  Disaster, war and conflict  Stresses of cultural assimilation for migrant or ethnic groups  Being LGBT  Discrimination  Trauma, abuse or bullying  Incarceration (particularly in the early stages)  **Society and Health Systems Level**  Access to means  Inappropriate media reporting  Stigma associated with help-seeking behaviour  **Risk factors for Children and Young People**  Family factors such as mental illness, substance misuse, & instability.  Abuse (emotional, physical & sexual), neglect, & witnessing domestic violence. Academic pressures, especially related to exams.  Bullying.  Bereavement, especially through suicide.  Previous self-harming.  Suicide-related internet use.  Being in care / looked after.  Being LGBT or having concerns about sexuality.  Social isolation or withdrawal.  Physical health conditions that have a social impact, eg asthma, acne.  Alcohol & illicit drug use.  Mental ill-health.  Relationship breakup. | Physical Illness  Financial difficulty & economic adversity  Harmful Gambling  Substance Misuse  Domestic Abuse  Going through divorce or separation  Social Isolation | Children & Young People  Middle aged Men  People who have self-harmed  People in contact with Mental Health services  People in contact with the justice system  Autistic People  Pregnant women and new mothers | **Children & Young People**  *(Changing risks and intervention points)*  Parents poor health  Deprivation  Bereavement  Bullying  Sexual & Physical Abuse  Social Isolation  Physical, neurodevelopmental and mental health issues  Adverse childhood experiences  Attachment and bonding  **Adolescents and Young Adults**  *(additional pressures and risks)*  Puberty  Sexuality  Exam Pressures  Work Pressures  Transitions between services  Adverse childhood experiences  Relationships  Leaving Home  Planning for future / career and education decisions  **Adults**  *(Difficult times)*  Work and financial pressures  Parenting responsibilities  Physical and Mental Health illnesses  Issues with substances  Relationship challenges  Women: Pregnancy, childbirth, menopause  Men: Stigma  **Older Adults**  Retirement, loss of work and reduced financial stability  Physical and neurological challenges  Social isolations and loneliness  Access to health support  Continued belonging to communities |
